# Regulatory T cells provide chondroprotection through increased TIMP1, IL-10 and IL-4, but cannot mitigate the catabolic effects of IL-1β and IL-6 in a tri-culture model of osteoarthritis

**DOI:** 10.1016/j.ocarto.2021.100193

**Published:** 2021-07-16

**Authors:** Laura E. Keller, Elia D. Tait Wojno, Laila Begum, Lisa A. Fortier

**Affiliations:** a Cornell University, College of Veterinary Medicine, Department of Clinical Sciences, USA; b University of Washington, Department of Immunology, USA

**Keywords:** Osteoarthritis, Regulatory T cells, T helper 17 ​cells, Interleukin 6, Interleukin 10, TIMP1

## Abstract

**Objective:**

To gain insight into Treg interactions with synovial tissues in early OA, an equine tri-culture model of OA was used to test the hypothesis that Tregs, in the absence of T Helper 17 ​cells, are sufficient to resolve inflammation elicited by IL-1β.

**Methods:**

To model normal and OA joints, synoviocytes were co-cultured with chondrocytes in a transwell system and ± stimulated with IL-1β. Tregs were activated and enriched, then added to co-cultures, creating tri-cultures. At culture end, synoviocytes and chondrocytes were analyzed for gene expression, Treg Foxp3 expression was reexamined by flow cytometry, and conditioned media were evaluated by ELISA.

**Results:**

Tregs increased IL-10 and IL-4 in tri-culture media and increased *TIMP1* gene expression in synoviocytes and chondrocytes. Tregs increased IL-6 in conditioned media and *Il6* gene expression in synoviocytes, which was additive with IL-1β. In chondrocytes, addition of Tregs decreased *Col2b* gene expression while *Acan* gene expression was decreased by IL-1β and addition of Tregs. IL-17A was detected in tri-cultures. CCL2 and CCL5 were increased in tri-cultures.

**Conclusions:**

In a tri-culture model of OA, addition of Tregs resulted in conditions conducive to chondroprotection including increased concentration of IL-10 and IL-4 in conditioned media and increased gene expression of *TIMP1* in both chondrocytes and synoviocytes. However, there was increased concentration of the catabolic cytokine IL-6, and decreased gene expression of *Col2b* and *Acan* in IL-1β-stimulated chondrocytes. These results suggest that blocking IL-6 could enhance Treg function in mitigating OA progression.

## 1 Introduction

In osteoarthritis (OA), immune cells from the peripheral blood infiltrate into the synovial membrane and synovial fluid of the affected joint [1]. Inflammatory cytokines and chemokines such as CCL2 and CCL5 are released from chondrocytes and synoviocytes with resultant chemotaxis of immune cells such as macrophages and T cells [2,3]. Activation and polarization of synovial macrophages contributes to inflammation, pain, and joint destruction [4]. T cells also regulate the OA disease process with infiltration of pro-inflammatory T Helper 17 (Th17) cells [5,6] and enrichment of anti-inflammatory Regulatory T (Treg) cells in the joint [7]. Because of their opposing roles in immunity and inflammation, an imbalance between pro-inflammatory Th17 and immunomodulatory Treg cells has been implicated in many autoimmune diseases including rheumatoid arthritis and psoriatic arthritis [8].

Tregs suppress proliferation, activation, and cytokine production by CD4^+^ T cells and CD8^+^ T cells to maintain immune homeostasis [9]. Tregs play a critical role in the maintenance of organismal homeostasis and are found to be tissue-resident in multiple tissues throughout the body including adipose tissue, skeletal muscle, and the colonic lamia propria [10]. Within skeletal muscle, Tregs promote tissue regeneration in the face of acute or chronic injury [11]. Tregs within synovial tissue may play a similar role in the absence of infection and autoimmunity as they accumulate in the synovial fluid and membrane of joints with early [12] and end-stage OA [7]. When activated, Tregs control magnitude and length of immune responses through several mechanisms. This includes cell-to-cell signaling through immune checkpoints CTLA-4 and PD-L1 and secretion of soluble mediators including IL-10, IL-35, and TGF-β1 [13]. The anti-inflammatory and anabolic cytokine IL-10 is strongly associated with Treg function [14]. IL-10 plays a role in the prevention of autoimmune disease by downregulating secretion of pro-inflammatory cytokines from effector T helper cells and reducing expression of co-stimulatory molecules on macrophages, and correct temporal release of IL-10 is critical for resolution of inflammation [15].

In chondrocytes, IL-10 treatment inhibits synthesis of IL-1β and TNF-α and suppresses proliferation and expression of NF-κB in cartilage collected from patients with end-stage OA [16]. In IL-1β-stimulated chondrocytes, IL-10 does not reduce *MMP13* [17], but it can increase TIMP1 secretion from IL-1β stimulated synoviocytes [18] providing a potential mechanism by which Tregs could restore metabolic balance to a joint. Animal models also support a role for Tregs and IL-10 in chondroprotection. Retroviral transduction of chondrocytes with IL-10 conveys protection from IL-1β-induced *ADAMTS4* but not *MMP13* gene expression [17]. In a rabbit model of post-traumatic OA, intra-articular injection of synoviocytes overexpressing IL-10 five days post-injury resulted in improved histological cartilage scores [19]. Data from human patients are consistent with these studies. Following acute anterior cruciate ligament tear, IL-10 is increased in synovial fluid, but then decreases as early as three months post-injury [20]. Also in early OA, Tregs are enriched within the synovial membrane compared to synovial fluid and blood [12], even compared to these compartments in end-stage OA [7].

Despite increasing knowledge about the dynamics of Tregs and the role of IL-10 and TGF-β1 in early and chronic OA, little is known about how the milieu of cytokines and chemokines secreted by Tregs affects chondrocyte and synoviocytes. Further, the reciprocal relationship is unknown, that is how an inflammatory articular environment affects Treg phenotype and function. Understanding how and why Tregs are ineffective at mitigating OA progression could reveal new insights into immunotherapeutics for OA. To address this knowledge gap, we used a novel *in vitro* model of OA based on a transwell co-culture system and tested the hypothesis that Treg anti-inflammatory function in the absence of pro-inflammatory Th17 ​cells is sufficient to resolve inflammation and catabolism elicited by IL-1β in an *in vitro* model of OA.

## 2 Materials and methods

### 2.1 Identification of native Treg population in equine blood

Equine blood was collected to a final concentration of 40 U/ml heparin with approval from the Institutional Animal Care and Use Committee (n ​= ​6; 6–12 years of age). Peripheral blood lymphocytes (PBL) were isolated as previously described [21] and analyzed for surface and intracellular expression of CD4, CD25 and Foxp3 by flow cytometry. Tregs were identified as Foxp3-expressing cells within the CD4^+^CD25^hi^ gate [22]. Fluorescence was measured using a BD FACSymphony A5 Cell Analyzer (BD, Franklin Lakes, NJ) and analyzed with Flowjo software (TreeStar, Inc, Ashland, OR) with fluorescence-minus-one controls.

### 2.2 *In vitro* enrichment and activation of Treg populations

**Fig. 1:**
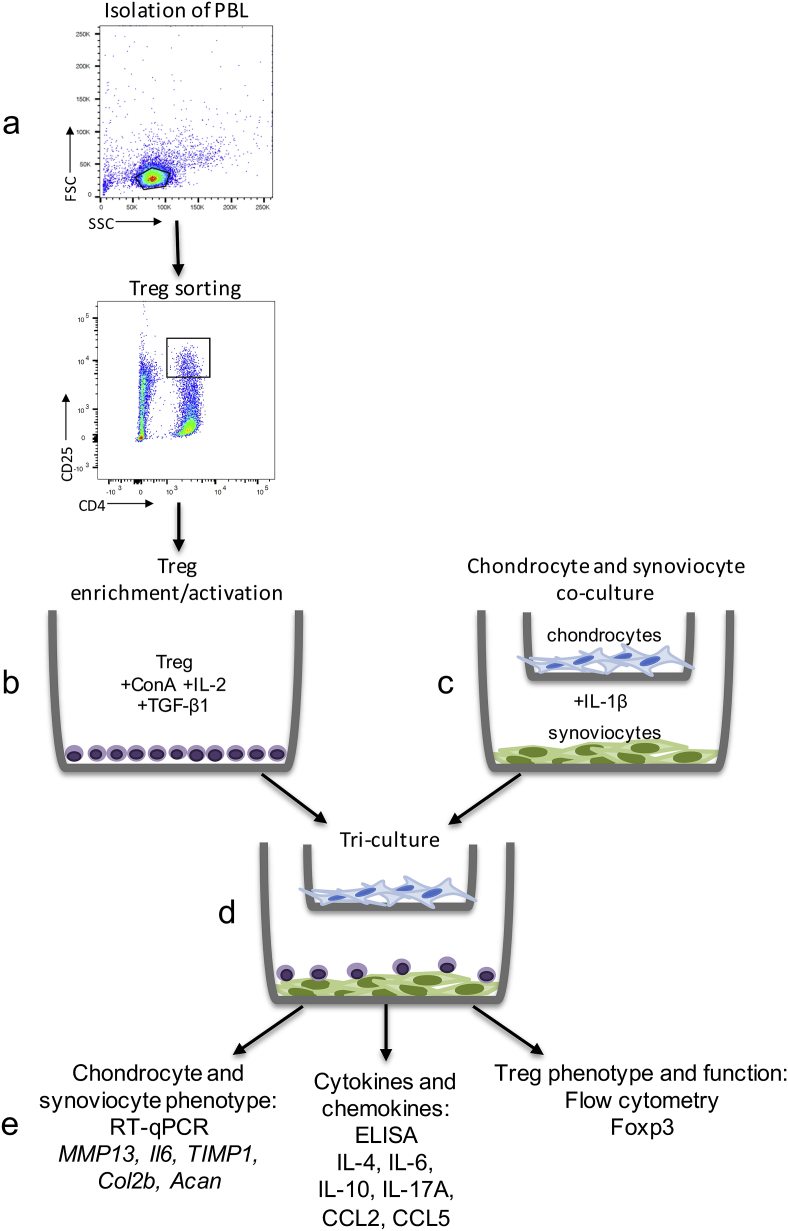
Study design overview. a) Peripheral blood lymphocytes (PBL) were isolated from equine (n ​= ​6) blood and Tregs were sorted using flow cytometric activated cell sorting. b) ConA, TGF-β1, and IL-2 were used to activate and differentiate Tregs and up-regulate protein expression of Foxp3 and secretion of anti-inflammatory cytokines. c) Simultaneously, synoviocytes/chondrocytes co-cultures were established and treated with IL-1β. d) Tregs were added in direct Treg-synoviocyte contact to create tri-cultures. e) Analysis of gene expression of catabolic (*MMP13*, *IL-6*) and anabolic (*TIMP1*, *Col2b*, *Acan*) genes was performed. Cytokines (IL-4, IL-6, IL-10, IL-17A) were analyzed in conditioned media samples. Flow cytometry was used to characterize Treg phenotype (Foxp3) to assess phenotype stability.

To obtain equine Tregs for *in vitro* studies of joint homeostasis, CD4^+^CD25^hi^ cells, which includes populations of Treg and effector T cells, were sorted (from the same 6 horses as above) using flow cytometry (Fig. 1a) and further differentiated into activated Tregs (Fig. 1b). Differentiation/activation was achieved by treatment of CD4^+^CD25^hi^ cells with concanavalin A (conA, 5 ​μg/mL; Sigma-Aldrich), rHu TGF-β1 (2 ​ng/mL; R&D Systems, Minneapolis, MN), and rHu IL-2 (100 U/mL; Peprotech, London, UK) as previously described [22] in modified RPMI medium containing 10% fetal bovine serum (FBS), 0.1 ​mM 2-mercaptoethanol, penicillin (100 U/mL), streptomycin (100 ​μg/mL) and basic fibroblastic growth factor (bFGF, 1 ​ng/mL) (Fig. 1c) [21]. Medium was replenished after day three, and cells were harvested at day six for addition to co-cultures as described below. Flow cytometry was also performed for Foxp3 to verify differentiation of CD4^+^CD25^hi^ cells into Tregs.

### 2.3 Co- and tri-cultures

Co-cultures of P0 chondrocytes and P2 synoviocytes from a single donor horse (1 year of age) were established with synoviocytes on the bottom of the tissue culture well and chondrocytes on the membrane insert (Fig. 1d) (pore size 0.4 ​μm; Millipore, Burlington, MA). Chondrocytes were kept at P0 to maintain phenotype and avoid dedifferentiation [23], and synoviocytes were passaged twice to reduce presence of synovial macrophages [24] to avoid MHC II interactions with non-matched Tregs. Additionally, use of cryopreserved chondrocytes and synoviocytes allowed for use of a single donor horse to reduce donor-to-donor variability. Co-cultures were maintained for 24 ​h in DMEM containing 10% FBS, 25 ​mM HEPES, ascorbic acid (50 ​μg/mL), α-ketoglutaric acid (30 ​μg/mL), L-glutamine (300 ​μg/mL), penicillin (100 U/mL), and streptomycin (100 ​μg/mL). Co-cultures were treated with or without rEq IL-1β (10 ​ng/mL; R&D Systems) for 24 ​h, washed with PBS, and medium was replenished without IL-1β. Intraarticular injection of IL-1β is used to model OA in horses by inducing synovitis and MMP activity [25], and GAG loss at a dose of 10 ​ng/mL [26].

To establish tri-cultures the *in vitro* enriched and activated Tregs were plated in direct contact with the synoviocytes in co-culture (Fig. 1e). After 24 ​hours, conditioned media samples were collected and Tregs were washed off the synoviocytes and centrifuged at 400×*g* for 5 ​minutes to pellet Tregs and clear conditioned media samples of cells and debris. Synoviocyte cultures were then observed by light microscopy to confirm removal of Tregs from synoviocyte surface. Conditioned media samples were stored at −80 ​°C for subsequent chemokine and cytokine analyses. Tregs were washed with PBS/BSA, then fixed and stained for Foxp3 (Supplemental Methods). Total RNA was isolated from synoviocytes and chondrocytes for gene analysis.

### 2.4 Outcome analyses

In chondrocytes and synoviocytes, expression of genes involved in joint homeostasis were quantified by RT-qPCR (Fig. 1f) using equine-specific primers and probes (Supplemental Methods, Table 1). Total gene copy number was determined using absolute quantitative PCR derived from a standard curve used for each gene at time of analysis and were normalized to 18S.

Cytokines and chemokines in the conditioned media were measured using multiplex assays for equine cytokines (IL-4, IL-10 and IL-17A) and chemokines (CCL2, CCL5) as previously described [27]. Concentrations of IL-6 were also measured according to manufacturer directions (R&D Systems, Minneapolis, MN).

### 2.5 Statistical analyses

Gene expression and cytokine/chemokine concentrations in conditioned media were analyzed using a generalized linear model with horse as a random effect. To compare cytokine concentration and Foxp3 expression pre- and post-Treg differentiation, a paired Wilcoxon non-parametric test was used. Tukey's post-hoc was used with p values ​≤ ​0.05 were considered significant. Statistical analyses were performed using JMP Pro 15 (SAS Institute, Cary, NC).

### 2.6 Cell staining for flow cytometric identification of Tregs

Isolated PBL were labeled with goat anti-human CD25 (R&D Systems) at 4° for 30 ​min, followed by donkey anti-goat immunoglobulin G-Phycoerythin (Invitrogen, Carlsbad, CA) as a secondary antibody, and mouse anti-equine CD4 (Washington State University, Pullman, WA) conjugated to Alexa Fluor 488 (Invitrogen). For extracellular staining and wash steps, phosphate-buffered saline (PBS) containing 0.5% bovine serum albumin (BSA) (Sigma-Aldrich, St. Louis, MO) and 0.02% sodium azide (VWR, Radnor, PA) (PBS/BSA) was used. Cells were then fixed, permeabilized and stained for Foxp3 using the eBioscience Foxp3 Transcription Factor Staining Buffer Set (eBioscience, San Diego, CA) per the manufacturer's instructions. Cells were stained with rat anti-mouse Foxp3 eFluor 450 (eBioscience) at 4° for 30 ​min.

## 3 Results

### 3.1 Treg differentiation and activation

Enrichment of Tregs was confirmed by increased expression of Foxp3 in the CD4^+^CD25^hi^ population. In the stimulated group, 79 ​± ​3% of CD4^+^CD25^hi^ cells expressed Foxp3 compared to 47 ​± ​5% in the naïve unstimulated population (p ​< ​0.0001). (Supplemental Figure 1).

### 3.2 Chondrocyte and synoviocyte responses to Tregs

**Fig. 2:**
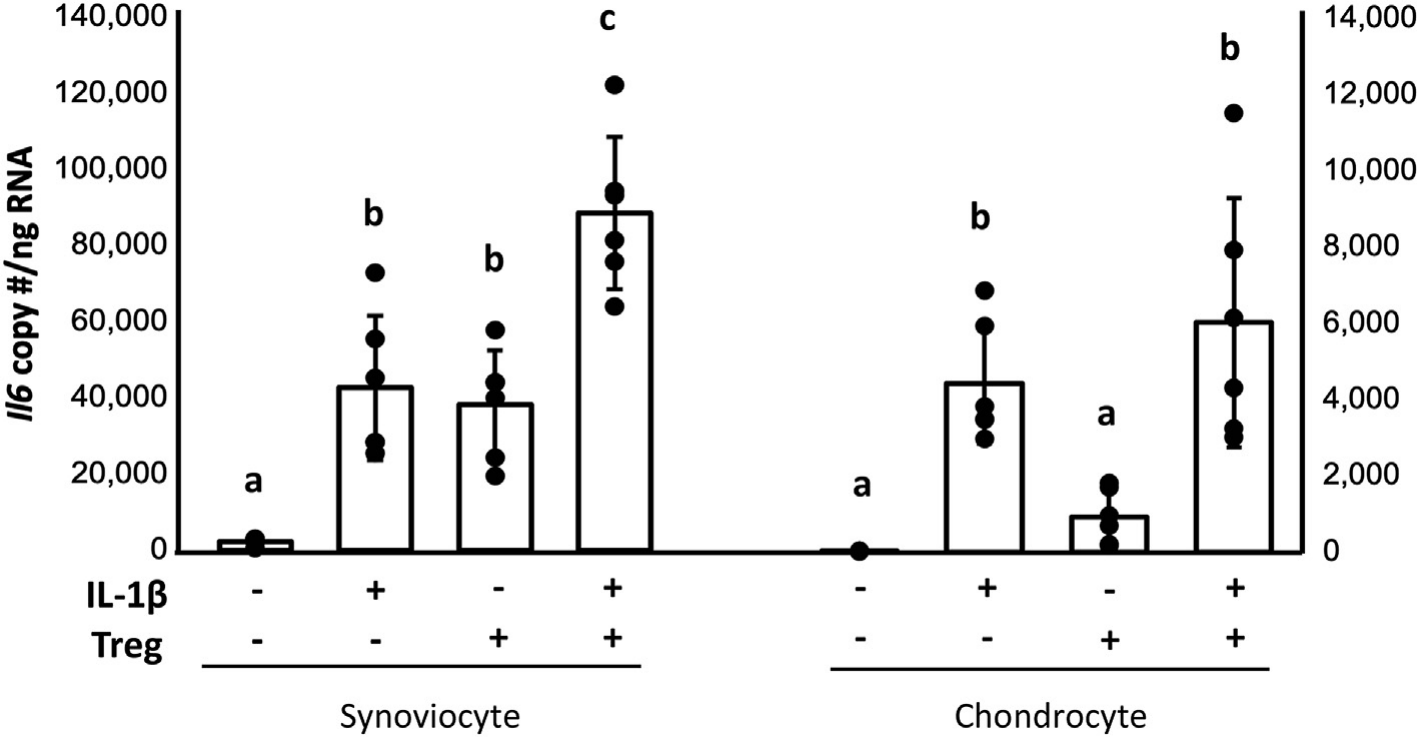
IL-1β or Tregs alone increased *Il6* in synoviocytes and appeared to act synergistically on *Il6* gene expression. Tregs did not similarly increase *Il6* in chondrocytes either alone or following stimulation with IL-1β. GLM with Tukey's post-hoc, groups that do not share a letter are statistically different, p ​< ​0.05.

**Fig. 3:**
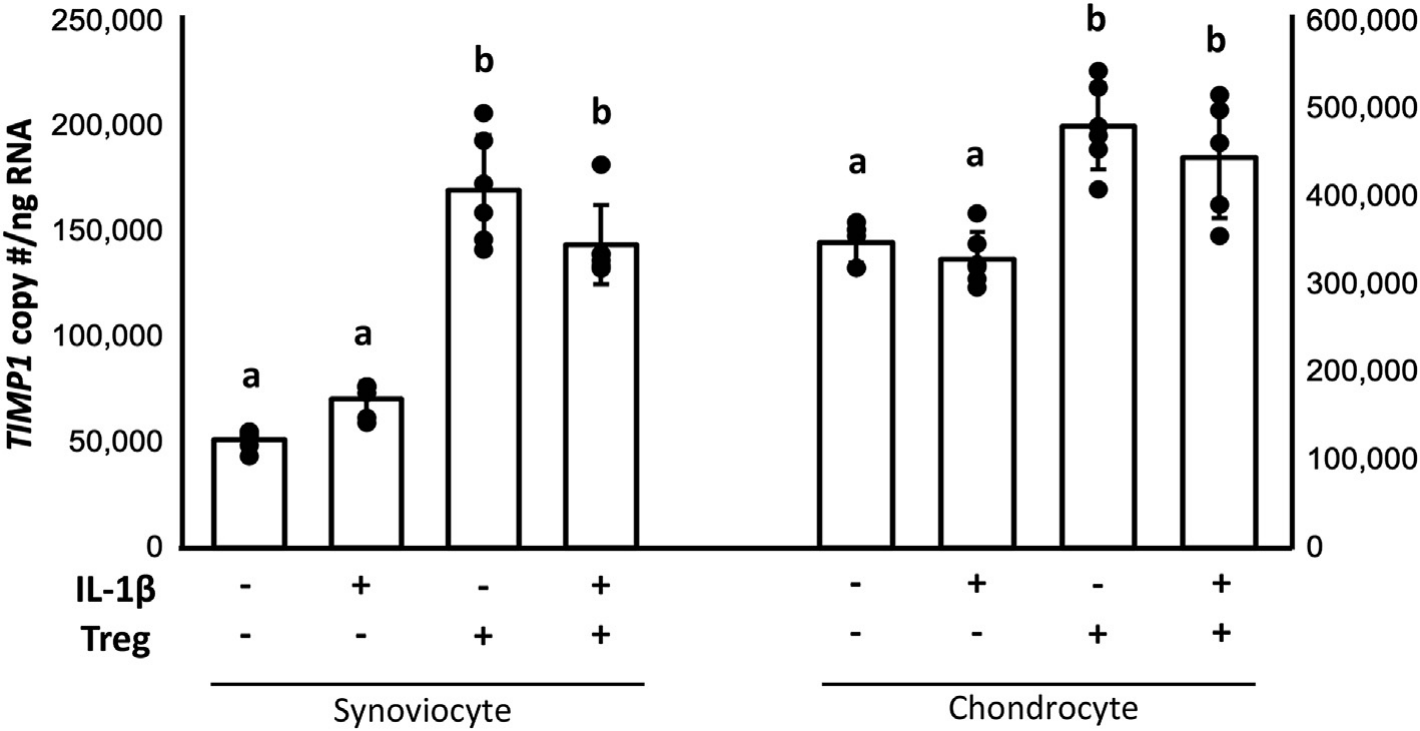
Treg soluble factors promoted *TIMP1* gene expression in both synoviocytes and chondrocytes regardless of stimulation with IL-1β. *TIMP1* was increased in synoviocytes in both tri-culture groups as Tregs promoted restoration of catabolic imbalance. Chondrocytes followed the same trend, suggesting that increased *TIMP1* gene expression could be contributed to factors within the Treg secretome. GLM with Tukey's post-hoc, groups that do not share a letter are statistically different, p ​< ​0.05.

*Pro- and anti-inflammatory and catabolic gene expression in synoviocytes and chondrocytes* - As expected, addition of IL-1β to co-cultures increased gene expression of *MMP13* in synoviocytes (p ​= ​0.02) and chondrocytes (p ​= ​0.0002) compared to controls (Supplemental Figure 2). Addition of Tregs did not affect gene expression of *MMP13* in IL-1β-stimulated synoviocytes (p ​= ​0.18) or chondrocytes (p ​= ​0.35). Gene expression of *Il6* was similarly increased by IL-1β in synoviocytes (p ​= ​0.001), and chondrocytes (p ​= ​0.005) (Fig. 2). Surprisingly, addition of Tregs in the absence of IL-1β also increased gene expression of *Il6* in synoviocytes (p ​= ​0.0025), but not chondrocytes (p ​= ​0.80). Tregs and IL-1β together had an additive effect on *Il6* gene expression compared to IL-1β alone in synoviocytes (p ​= ​0.0002), but not chondrocytes (p ​= ​0.50), suggesting independent mechanisms for stimulation of *Il6* gene expression by IL-1β and Tregs. Gene expression of the matrix-sparing glycoprotein *TIMP1* was increased when Tregs were added to co-cultures. *TIMP1* was increased in synoviocytes (p ​< ​0.0001) and chondrocytes (p ​< ​0.03) in the presence or absence of IL-1β, suggesting a possible mechanism for mitigating extracellular matrix catabolism (Fig. 3).

**Fig. 4:**
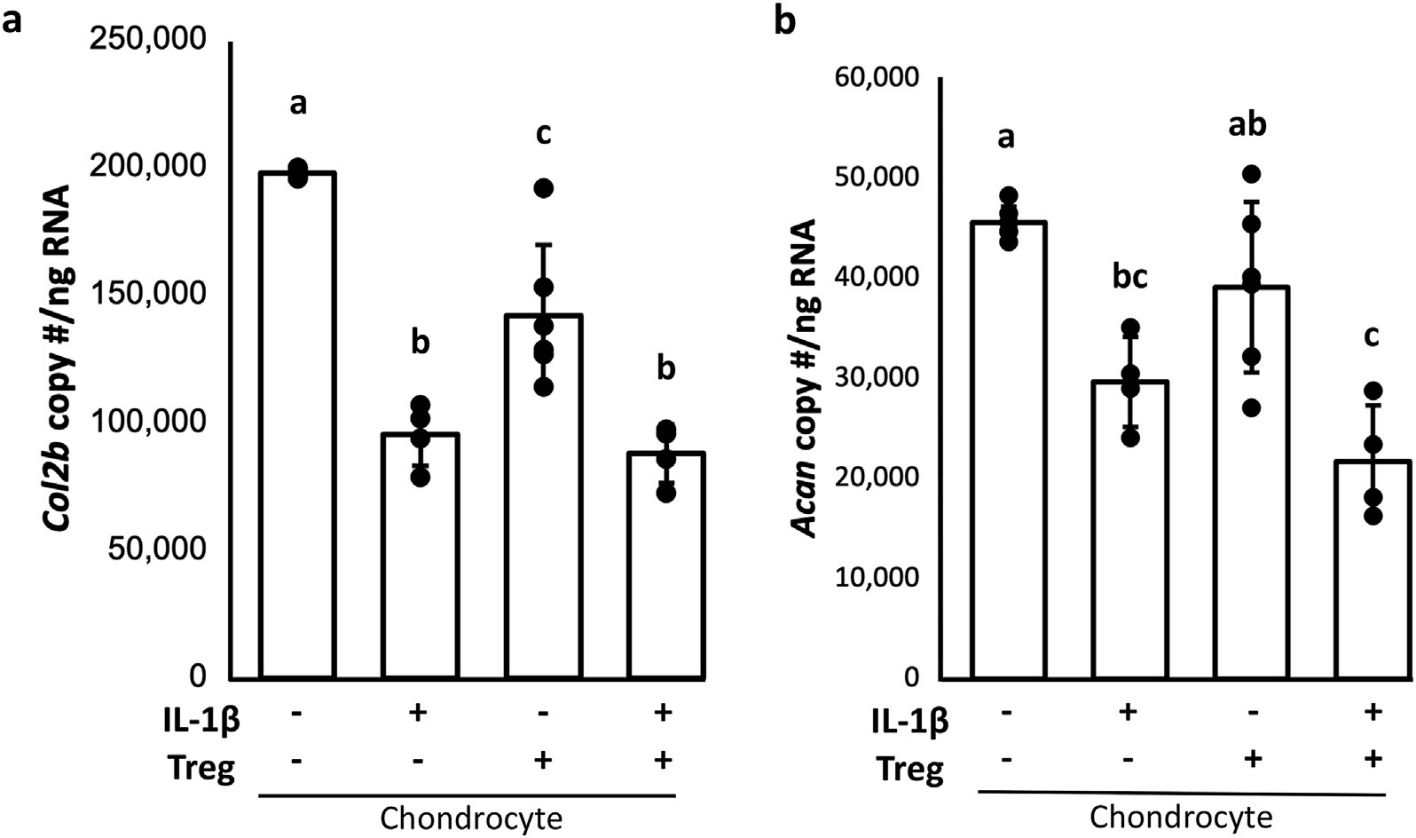
Tregs alone or in combination with IL-1β decrease expression of structural protein in cartilage a) Tregs alone decreased gene expression of *Col2b* in chondrocytes despite the presence of IL-10 and IL-4. b) Addition of Tregs to IL-1β-stimulated chondrocytes further decreased gene expression of *Acan*, promoting degradation of cartilage tissue. GLM with Tukey's post-hoc, groups that do not share a letter are statistically different, p ​< ​0.05.

*Matrix gene expression in chondrocytes* – Gene expression of *Col2b* was decreased by IL-1β (p ​< ​0.0001), and to a lesser extent by Tregs alone in the absence of IL-1β (p ​= ​0.0002) (Fig. 4a). The combination of IL-1β and Tregs was not additive and resulted in decreased *Col2b* gene expression to a level not different from IL-1β alone (p ​= ​0.9). For *Acan,* gene expression was unchanged by the addition of Tregs alone (p ​= ​0.013) (Fig. 4b). In cultures treated with Tregs in addition to IL-1β, gene expression was decreased compared to control cultures (p ​= ​0.0005), but was not different from IL-1β treated cultures (p ​= ​0.30).

### 3.3 Secretion of cytokines and chemokines and growth factors into tri-culture conditioned media

**Fig. 5:**
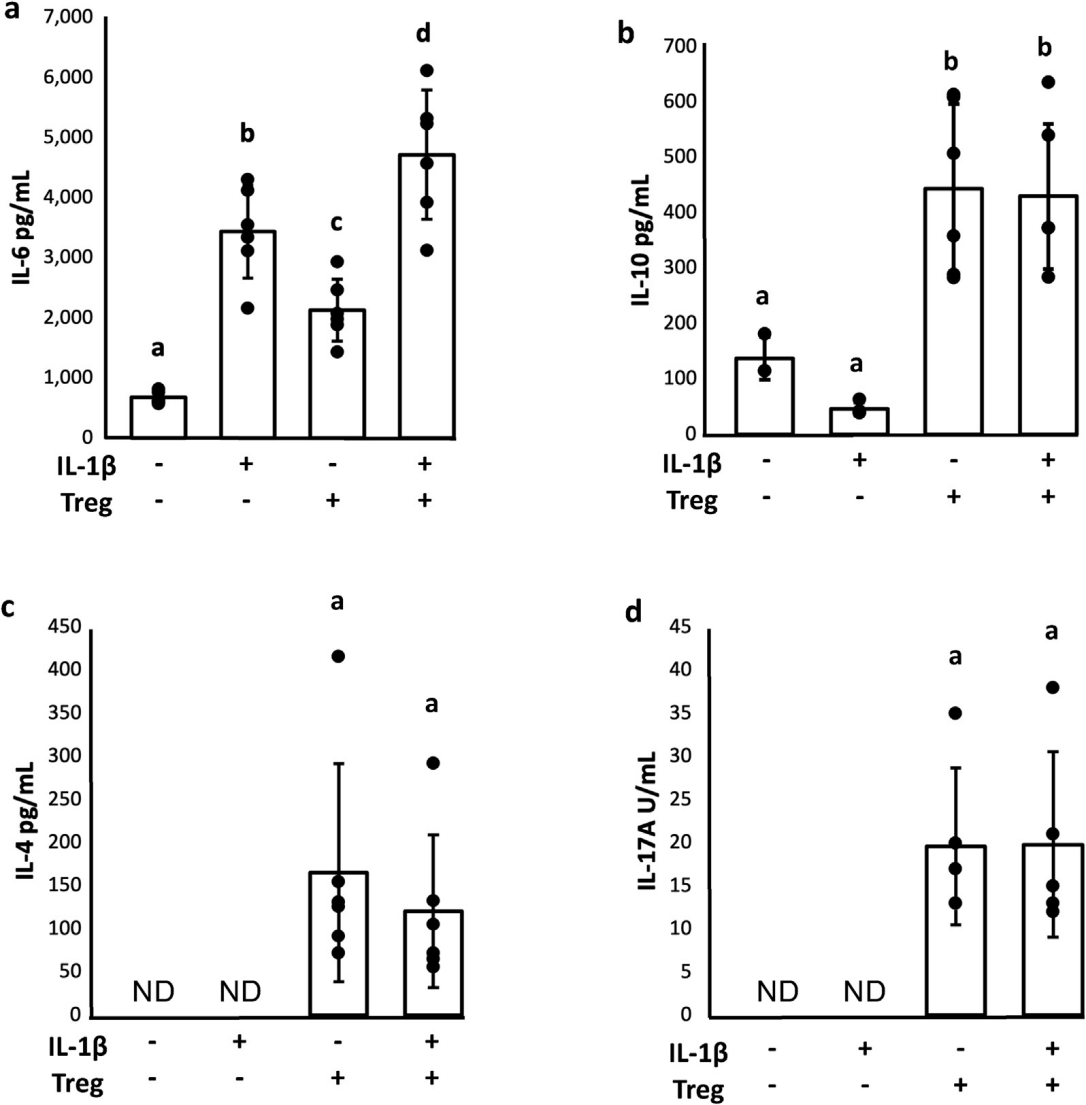
Tregs simultaneously increase concentrations of chondroprotective and anti-inflammatory cytokines in conditioned media samples while promoting secretion of pro-inflammatory IL-6 by chondrocytes and synoviocytes. a) IL-6 concentrations followed trends of *Il6* gene expression in synoviocytes, confirming its secretion following upregulation of gene expression. b) Enriched Tregs secreted high, unchanged concentrations of IL-10, regardless of inflammatory environment. c) Residual Th2 cells within the enriched Treg population secreted IL-4 in tri-cultures. d) IL-17A was detected in conditioned media samples, but inflammatory environment did not promote its secretion by T cells. GLM with Tukey's post-hoc, groups that do not share a letter are statistically different, p ​< ​0.05. ND ​= ​not detected.

*Cytokines IL-6, IL-10, IL-4, and IL-17A -* Protein concentration of the catabolic cytokine IL-6 was increased in a similar, but not identical pattern to IL-6 gene expression in chondrocytes and synoviocytes. Both IL-1β (p ​< ​0.0001) and Treg-stimulated co-cultures (p ​= ​0.007) had increased IL-6 in the conditioned media samples compared to co-culture controls (Fig. 5a). Tregs did not increase protein secretion of IL-6 to the same extent as IL-1β stimulated cultures. Similar to gene expression, the effects of IL-1β and Tregs appear to be additive (p ​< ​0.0001).

The anabolic cytokine IL-10 is indicative of Treg function [14]. The addition of Tregs significantly increased IL-10 in conditioned media samples and was not affected by IL-1β treatment (Fig. 5b; p ​< ​0.001). This suggests that Treg were phenotypically functioning and stable in the conditions of this study. Th2 cells characteristically secrete IL-4 which was significantly increased in cultures with Tregs and unaffected by the addition of IL-1β (Fig. 5c). Interestingly IL-4 was below the limit of detection in co-cultures with no Tregs present. Like IL-4, IL-17A, which is secreted by Th17 effector cells, was Treg-dependent and detected in tri-cultures, but not co-cultures, and was unaffected by IL-1β (Fig. 5d).

**Fig. 6:**
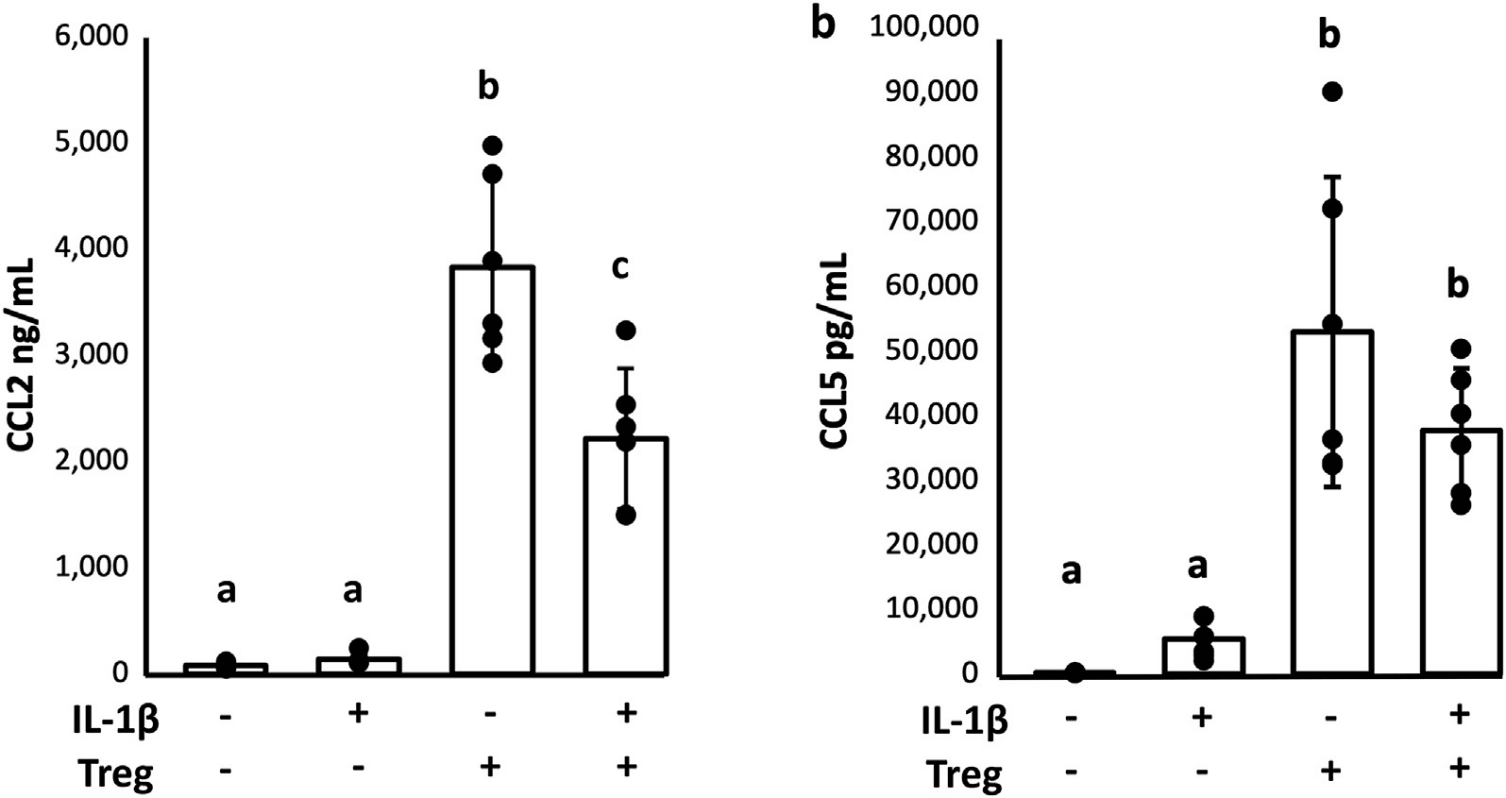
Tregs secrete high concentrations of macrophage and T cell chemoattractants. a) CCL2 concentration was not affected by IL-1β stimulation alone. Addition of Tregs increased CCL2 which was then diminished when both IL-1β and Tregs were in the tri-cultures. b) CCL5 was similarly unaffected by IL-1β alone and significantly increased with the addition of Tregs. Addition of IL-1β did not decreased Treg-induced CCL5 concentration. GLM with Tukey's post-hoc, groups that do not share a letter are statistically different, p ​< ​0.05.

*Chemokines CCL2 and CCL5* – In OA, CCL2 and CCL5 are increased in synovial fluid and thought to be secreted by injured chondrocytes and synoviocytes. Both CCL2 and CCL5 were present at very low concentrations in co-cultures and significantly increased tri-cultures suggesting that activated Tregs were the main source of these chemokines (Fig. 6). CCL2, but not CCL5 was decreased in tri-cultures treated with IL-1β.

### 3.4 Treg phenotype and function after tri-culture

At culture end, Tregs were removed and Foxp3 expression was reassessed. Pre-culture, Foxp3 expression was (79 ​± ​3%) and significantly decreased in both control (66 ​± ​4%; p ​< ​0.0001) and IL-1β-treated (65 ​± ​3%; p ​< ​0.0001) tri-cultures which were not different from each other (p ​= ​0.98).

## 4 Discussion

The purpose of this study was to determine if Tregs alone were sufficient to mitigate inflammation and matrix catabolism in an *in vitro* model of early OA. A tri-culture model was used to study the effects of Tregs on chondrocytes and synoviocytes, and to study the reciprocal relationship of how Treg phenotype and function are affected by joint inflammation. Our hypothesis was that Treg anti-inflammatory function would be sufficient to resolve inflammation and catabolism elicited by IL-1β. The use of equids to establish a tri-culture system allowed for sufficient collection of Tregs from peripheral blood and matched, cryopreserved chondrocytes and synoviocytes, which would not have been possible if using a small animal or human model. Further, the horse is an established model for OA with cartilage thickness and biomechanical loading approximating that of humans [28,29].

A highly enriched Treg population failed to completely restore homeostasis in IL-1β-treated chondrocytes or synoviocytes. Either incomplete differentiation of residual effector T cells that continued secreting pro-inflammatory cytokines or Treg instability could have been a contributing factor. The T cell population at the beginning of the experiment was 72–92% Tregs after differentiation based on Foxp3 expression. In conditioned media samples, detection of IL-4 and IL-17A suggests that residual Th2 (IL-4) and Th17 (IL-17A) cells were present in the Treg-enriched population used in this study, or that some Tregs demonstrated phenotypic instability. A varied T cell phenotype is a more realistic representation of cellular infiltration into the joint during OA than a pure Treg population. Even if a pure Treg population could have been achieved, there was evidence that Treg phenotype was not stable during culture duration. At study end, about 10% fewer cells were expressing Foxp3 in both IL-1β-stimulated and control tri-cultures, suggesting destabilization in Treg function. Treg destabilization, wherein the presence of pro-inflammatory factors, including IL-6, cause Tregs to lose Foxp3 expression and suppressive functions in favor of a pro-inflammatory phenotype, has been previously described [30]. The reduction in Foxp3 expression following tri-culture was surprising, as it has previously been reported that induced Tregs maintain immunosuppressive phenotype and function when interacting with TNF-α-treated synoviocytes from rheumatoid arthritis patients undergoing knee arthroscopy or synovectomy [31]. Differences in induced Treg stability between experiments could have been a result of variations in synoviocyte populations and stimulation methods or that, in those experiments, Tregs were continually stimulated with anti-CD3/CD28-coated beads, simulating interactions with antigen-presenting cells in T cell activation, whereas in the experiments of this study, Tregs did not receive continuous ConA stimulation during tri-culture.

Tregs were unable to alter IL-1β-induced *MMP13* gene expression in either chondrocytes or synoviocytes. MMP13 is well known as an extracellular matrix-degrading enzyme and is highly expressed in synovium and cartilage of OA joints and is therefore studied as a biomarker and potential target for OA treatment [32]. Unlike other MMPs, MMP13 can cleave intact type 2 collagen *in vivo* [33], and decreases gene expression of *Col2a* and *Acan in vitro* [34] so the inability of Tregs to affect *MMP13* gene expression is a significant shortcoming in the concept of Tregs as a target or Treg secretome as a treatment for OA. The extracellular activity of MMPs is specifically antagonized by TIMPs including TIMP1 [35]. *TIMP1* gene expression was increased by addition of Tregs in both chondrocytes and synoviocytes even in the presence of IL-1β. However, TIMP1 alone is not sufficient for chondroprotection as shown by addition of exogenous TIMP1 to IL-1β stimulated chondrocytes that resulted in decreased MMP3 concentration but did not protect against matrix catabolism [36]. Similarly, in the present study, despite an increase in *TIMP1* gene expression in Treg-containing cultures, neither *Col2b* nor *Acan* expression were protected from IL-1β.

Detection of IL-4 in tri-culture conditioned media samples was unexpected. IL-4 is secreted by Th2 cells and plays a critical role in wound healing by promoting alternate activation of macrophages to an anti-inflammatory M2 phenotype [37]. Within the context of OA, IL-4 alone or in combination with IL-10 protects cartilage in a dose-dependent manner by rescuing cartilage proteoglycan synthesis and release, and reducing secretion of IL-1β and TNF-α by cartilage explants exposed to whole blood [38]. However, a subsequent study by the same group revealed that IL-4/IL-10 administration into joints of hemophilic mice following joint bleeds did not prevent an increase in cartilage OARSI score following Safranin-O Fast-Green staining, nor did it prevent an increase in synovial inflammation as determined by Valentino visual bleeding score. It was suggested that failure of IL-4/IL-10 administration to control inflammation within this context was the short half-life of these two molecules (<2 ​h after intravenous injection). However, continuous secretion of IL-4 and IL-10 by activated Tregs within the tri-cultures would suggest there is another mechanism leading to failure of these cytokines to prevent IL-1β-induced decrease in *Col2b* and *Acan* in chondrocytes.

The chondroprotective effects of IL-4 and IL-10 may also have been negated by IL-6, which was increased by IL-1β and Tregs alone, and further when in combination. IL-6 enhances synthesis of MMP13 in synoviocytes and chondrocytes [39] while suppressing collagen type II and aggrecan synthesis in chondrocytes [40]. In synovial fluid and serum from patients with OA, IL-6 concentration correlates with disease severity [41]. Secretion of IL-6 into the conditioned media samples of the present study may be responsible for the failure of Tregs to reduce *MMP13* gene expression in chondrocytes and synoviocytes and protect *Col2b* and *Acan* gene expression in chondrocytes. Moreover, IL-1β increases expression of membrane-bound IL-6 receptor on chondrocytes, which may explain why Tregs do not rescue *Col2b* gene expression and appear to have a synergistic effect with IL-1β on the decrease in *Acan* gene expression. Increased IL-6 concentration in conditioned media samples of Treg-containing cultures was unexpected. If IL-6 within this tri-culture system is responsible for the suppression of Treg anti-inflammatory functions, then neutralizing IL-6 or blocking its receptor could remove the inhibitory effects of IL-6 on Treg functions. Anti-IL-6 therapy has been met with success in the treatment of rheumatoid arthritis, and mice treated with anti-IL6 and/or anti-IL-6R antibody therapy markedly reduces post-traumatic OA, laying the foundation for its use as a therapy to treat OA [41,42]. If Tregs alone did not lead to the increase in IL-6 in control tri-culture, residual Th17 ​cells may have promoted increased IL-6 secretion by synoviocytes through IL-17A [43].

IL-17A was detected in cultures where Tregs were present and was independent of IL-1β. IL-17A leads to joint catabolism through increased synthesis of MMP1 and MMP13 by chondrocytes and synoviocytes [5]. The most likely source of IL-17A was residual effector Th17 ​cells remaining in the CD4^+^CD25^hi^ population rather than a result of Treg phenotype plasticity in the inflammatory environment. Concentrations of IL-10 and IL-17A were unchanged in tri-cultures treated with IL-1β suggesting that, although there was a decrease in Foxp3^+^ Tregs following tri-culture, this was not due to plasticity resulting from promotion of a Th17 phenotype despite the OA environment of the IL-1β-stimulated tri-culture.

Concentrations of CCL2 and CCL5 were considerably higher in tri-cultures compared to co-cultures and CCL2, but not CCL5, was significantly decreased in tri-cultures stimulated with IL-1β. CCL2 recruits monocytes and T cells to sites of injury as part of a normal acute inflammatory response. In a mouse model of OA, the CCL2/CCR2 axis was shown to be involved in the recruitment of pro-inflammatory macrophages to the inflamed joint [44]. In CCL2 knockout mice, M2 macrophages were nearly absent compared to controls, further demonstrating a role for CCL2 in maintaining an M1/M2 balance [45]. CCL2 released by Tregs in the tri-cultures of the present study would not only act as a powerful monocyte chemoattractant but could also maintain homeostasis of M1/M2 phenotype of monocytes recruited to the joint. The CCL5/CCR5 axis is involved in T cell migration and recruitment, and maintenance of M2 phenotype in tumor-associated macrophages [46]. CCL5 is secreted by several cell types, including T cells following activation [47] and fibroblasts when stimulated with IL-1β [48]. CCL5 is also secreted by tumor cells in order to recruit Tregs to suppress pro-inflammatory effector T cells within the tumor microenvironment [49]. Secretion of CCL5 within the tri-culture by Tregs could be an attempt to recruit additional Tregs to the inflamed joint environment to suppress inflammation, as well as maintain or modify M1/M2 balance within the joint. The failure of Tregs to suppress IL-1β-induced increase in *MMP13* in synoviocytes and chondrocytes and prevent decrease of *Col2b* and *Acan* in chondrocytes may be due to the requirement of an intermediate step, such as monocytes or macrophages, to elicit anti-inflammatory effects on tissues within the joint [50]. One of the limitations of this study is that synoviocytes were cultured specifically to reduce presence of synovial macrophages in order to avoid MHC II crosstalk with Tregs from non-matched donors.

## 5 Conclusion

An activated and enriched Treg population was not sufficient to mitigate IL-1β-induced inflammation and catabolism in synoviocytes and chondrocytes despite secretion of anti-inflammatory cytokines IL-4 and IL-10. This indicates that either Tregs alone are not sufficient to restore joint homeostasis, or that the additional inflammation induced by IL-6 or IL-17A inhibited Tregs from restoring homeostasis within the tri-culture. Considering the results of this study, IL-6 is likely a contributing factor to Treg failure to mitigate inflammation. Future directions will include reducing the function of IL-6 *in vitro* in order to determine if it is significantly contributing to failure of Tregs to mitigate OA.

## Author contributions

LEK designed the study, performed data collection, analyzed the data, and drafted the manuscript. EDTW and LAF contributed to study design, data interpretation, and manuscript preparation. LB participated in data collection and reviewed the manuscript. All authors approved the final version of the manuscript.

## Declaration of competing interest

None.

## Role of the funding source

These studies were funded by 10.13039/100000002NIH RO1 AR071394 and the Paula Kennedy-Harrigan fund.

## Appendix A Supplementary data

Table S1 Equine-specific Taqman ® primers and probes used in gene expression analysis with a Viia 7 Real-Time PCR System (Applied Biosystems, Foster City, CA). Table S1

Fig. S1 Treg activation and enrichment. **a)** Peripheral blood lymphocytes (PBL) were sorted based on expression of CD4 and CD25. **b**) Tregs, defined as CD4^+^CD25^hi^ in this native population account for 39–51% of this population based on Foxp3 expression. **c)** Treg were analyzed again following incubation with ConA, rHu TGF-β1 and rHu IL-2 to confirm enrichment of the Foxp3^+^ Treg population. Fig. S1

Fig. S2 Addition of IL-1β increased expression of *MMP13* in synoviocytes and chondrocytes, but Tregs were not able to fully able to rescue this effect in synoviocytes and did not affect *MMP13* expression in chondrocytes. GLM with Tukey's post-hoc, groups that do not share a letter are statistically different, p ​< ​0.05. Fig. S2
