# Supplementary figures and images for: Regulatory T cells provide chondroprotection through increased TIMP1, IL-10 and IL-4, but cannot mitigate the catabolic effects of IL-1β and IL-6 in a tri-culture model of osteoarthritis
Source: Osteoarthr Cartil Open. 2021 Jul 16;3(3):100193. doi: 10.1016/j.ocarto.2021.100193 (PMC9718146; doi:10.1016/j.ocarto.2021.100193)

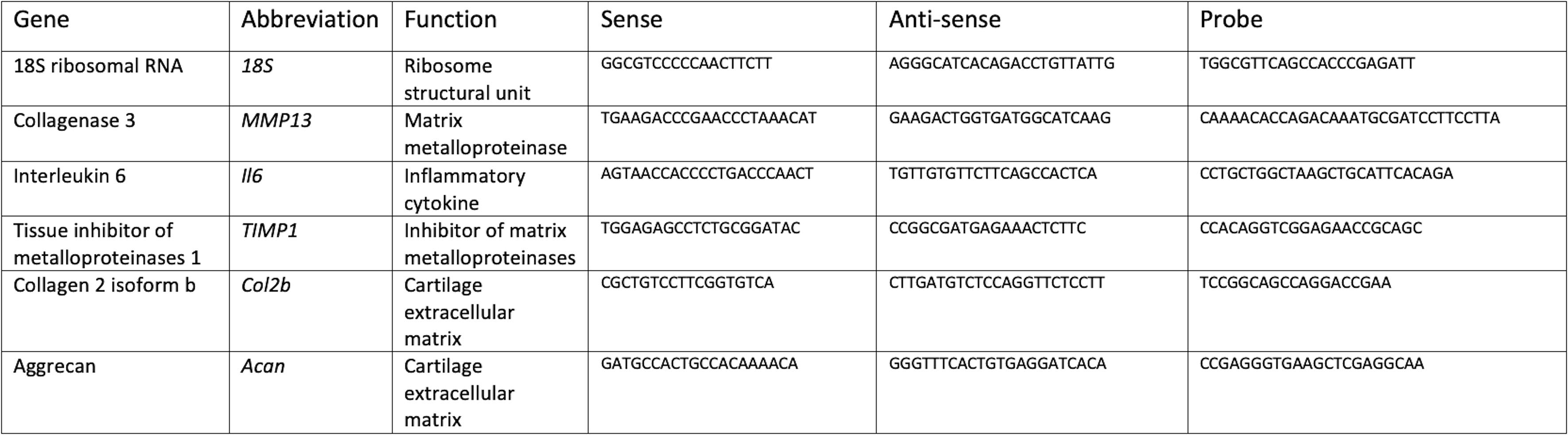

Supplement: Table S1 — Equine-specific Taqman ® primers and probes used in gene expression analysis with a Viia 7 Real-Time PCR System (Applied Biosystems, Foster City, CA). [file figs1.jpg]

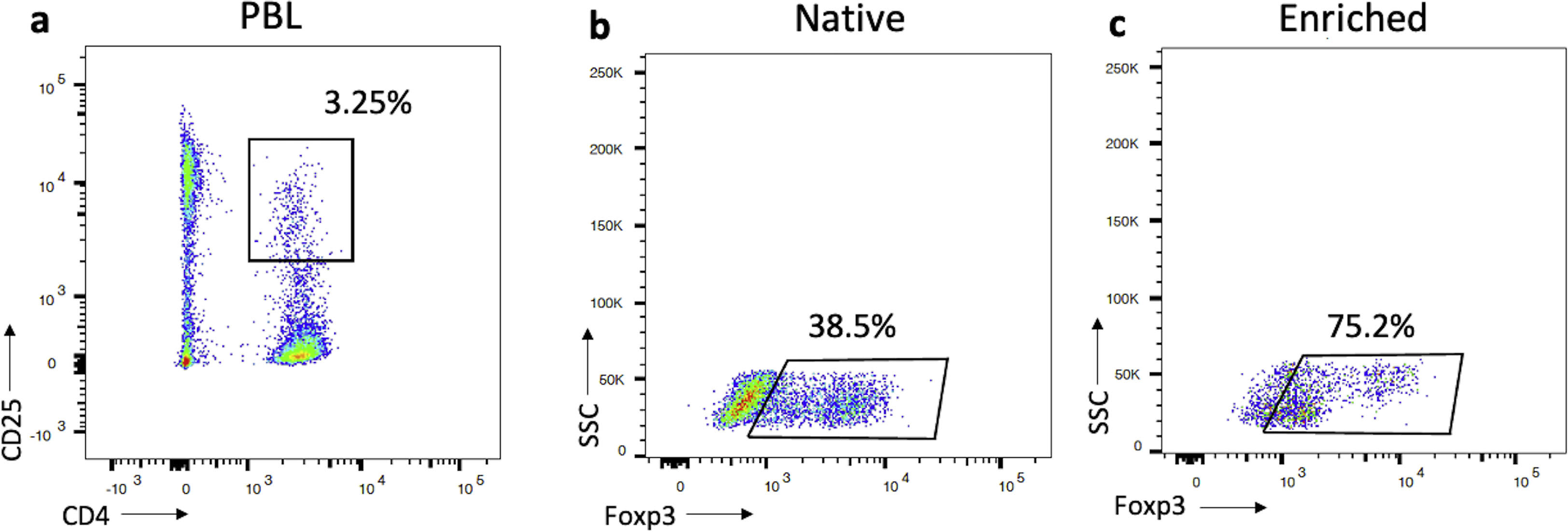

Supplement: Fig. S1 — Treg activation and enrichment. a) Peripheral blood lymphocytes (PBL) were sorted based on expression of CD4 and CD25. b) Tregs, defined as CD4+CD25hi in this native population account for 39–51% of this population based on Foxp3 expression. c) Treg were analyzed again following incubation with ConA, rHu TGF-β1 and rHu IL-2 to confirm enrichment of the Foxp3+ Treg population. [file figs2.jpg]

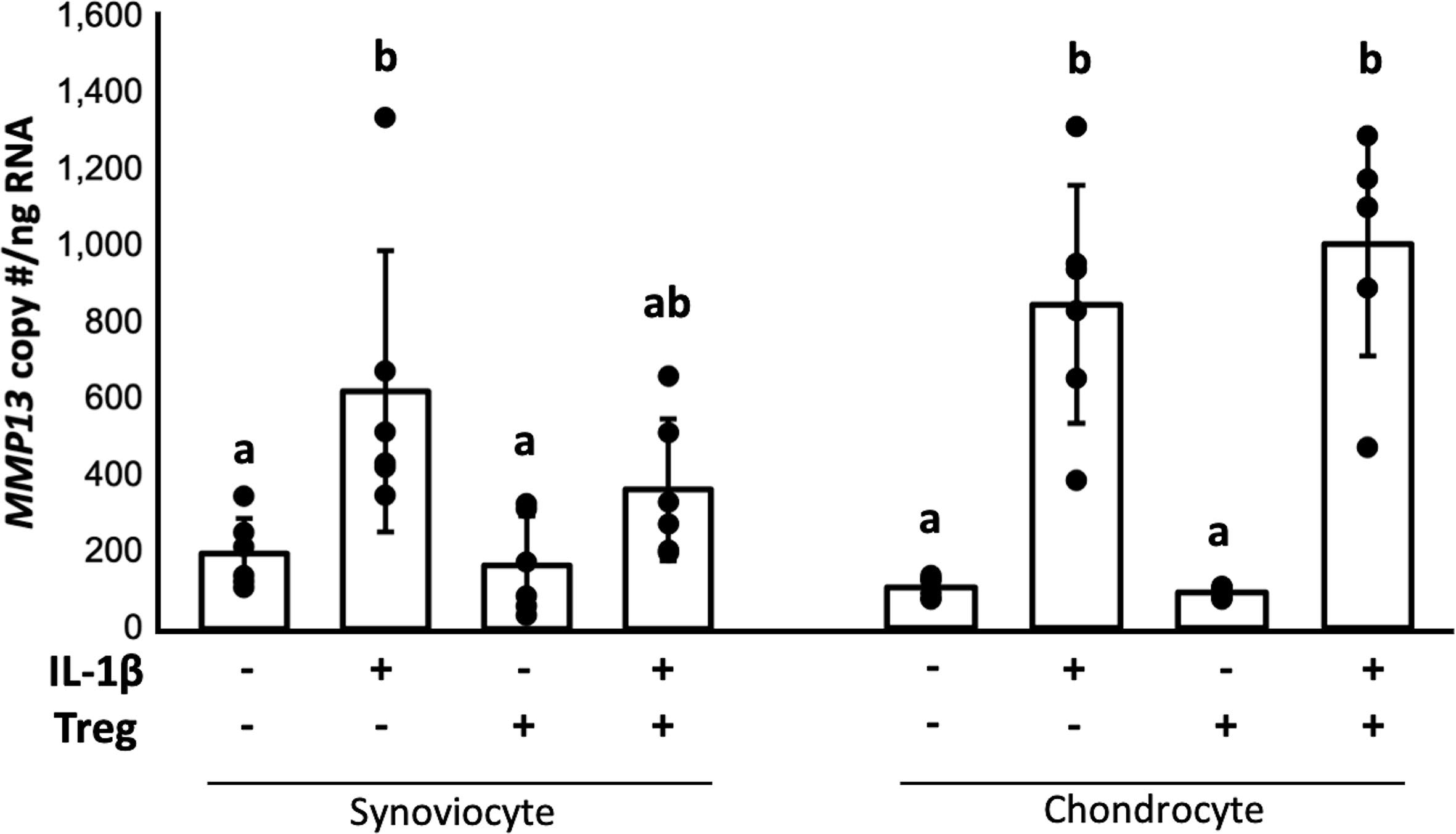

Supplement: Fig. S2 — Addition of IL-1β increased expression of MMP13 in synoviocytes and chondrocytes, but Tregs were not able to fully able to rescue this effect in synoviocytes and did not affect MMP13 expression in chondrocytes. GLM with Tukey's post-hoc, groups that do not share a letter are statistically different, p ​< ​0.05. [file figs3.jpg]
